# Engineered Interleaved Random Glass Fiber Composites Using Additive Manufacturing: Effect of Mat Properties, Resin Chemistry, and Resin-Rich Layer Thickness

**DOI:** 10.3390/polym15153189

**Published:** 2023-07-27

**Authors:** Ahmed M. H. Ibrahim, Mohanad Idrees, Emine Tekerek, Antonios Kontsos, Giuseppe R. Palmese, Nicolas J. Alvarez

**Affiliations:** 1 Department of Chemical and Biological Engineering, Drexel University College of Engineering, Philadelphia, PA 19104, USA; 2 Department of Mechanical Engineering and Mechanics, Drexel University College of Engineering, Philadelphia, PA 19104, USA

**Keywords:** additive manufacturing, interleaving, random chopped fibers, thermosetting polymers, interlaminar properties

## Abstract

Standard lay-up fabrication of fiber-reinforced composites (FRCs) suffer from poor out-of-plane properties and delamination resistance. While advanced manufacturing techniques (e.g., interleaving, braiding, and z-pinning) increase delamination resistance in FRCs, they typically result in significant fabrication complexity and limitations, increased manufacturing costs, and/or overall stiffness reduction. In this work, we demonstrate the use of facile digital light processing (DLP) technique to additively manufacture (AM) random glass FRCs with engineered interleaves. This work demonstrates how vat photo-polymerization techniques can be used to build composites layer-by-layer with controlled interleaf material, thickness, and placement. Note that this engineering control is almost impossible to achieve with traditional manufacturing techniques. A range of specimens were printed to measure the effect of interleaf thickness and material on tensile/flexural properties as well as fracture toughness. One important observation was the ≈60% increase in interlaminar fracture toughness achieved by using a tough resin material in the interleaf. The comparison between AM and traditionally manufactured specimens via vacuum-assisted resin transfer molding (VARTM) highlighted the limitation of AM techniques in achieving high mat consolidation. In other words, the volume fraction of AM parts is limited by the wet fiber mat process, and engineering solutions are discussed. Overall, this technique offers engineering control of FRC design and fabrication that is not available with traditional methods.

## 1. Introduction

Fiber-reinforced composite (FRC) materials consist of high-strength, high-modulus fibers embedded in a polymeric matrix. In an FRC, both fiber and matrix retain their physical and chemical identities, yet produce a combination of properties not obtainable by either component separately. Generally speaking, fibers act as load carriers and the matrix transfers the loads from fiber to fiber [1]. While FRCs offer significant advantages over metals, they have limitations. For example, glass fiber-reinforced composites (GFRCs) suffer from poor out-of-plane properties and tend to delaminate. The latter stems from a mismatch in properties between resin and fiber, i.e., significant differences in Poisson ratio, and mechanical/thermal properties, that lead to interlaminar normal/shear stresses and fiber delamination [2].

Significant effort has gone into enhancing the delamination resistance of FRCs, such as the use of toughened polymeric matrices [3], z-pinning [4], stitching [5], braiding [6], and optimizing stacking sequences [7]. Unfortunately, these ideas led to significant increases in fabrication costs and composite weight and/or loss of in-plane properties [2]. The most promising technique to reduce delamination is interleaving, which is the process of adding a discrete resin layer between consecutive fiber plies [8]. The interleaves, also known as resin-rich layers (RRLs), are either composed of a brittle or a ductile polymeric matrix. Interleaving has been experimentally proven to enhance modes I and II delamination resistance [9,10,11,12,13,14,15,16,17,18], as it leads to the creation of a plastic yield zone capable of absorbing a large amount of energy through plastic deformation [19,20].

One important drawback of RRL delamination resistance is the reduction in composite fiber volume fraction (FVF) with increasing RRL thickness, which inadvertently decreases mechanical properties. Furthermore, there is an optimum thickness of RRL whereby additional thickness does not offer any further delamination resistance. However, this thickness is not well known, but is argued to depend on the plastic zone length of the toughening resin [21]. The trade-off between delamination resistance and reduction in fiber volume fraction highlights the necessity of optimizing $\delta_{RRL}$ thickness.

Traditional fabrication techniques of interleaved composites are very limited to certain types of resins and typically are only achieved through pre-preg lay-up techniques. Furthermore, interleaving using traditional manufacturing techniques is labor intensive, limits the types of resins usable, and is of prohibitively high cost. The state-of-the-art would greatly benefit from an automated interleaving process, whereby a broad range of resins can be used, and additive manufacturing (AM) presents such an opportunity. AM techniques offer a novel approach to traditional manufacturing methods, allowing for multi-material manufacturing and geometric control and are attracting attention in a wide array of industries [22,23]. Recently, we have developed a method of producing GRFCs using digital light processing (DLP) stereolithography, a vat photo-polymerization AM technique utilizing liquid resins that cure selectively upon exposure to ultra violet (UV) light [21]. In a previous work, we showed that this technique is capable of producing GRFCs using woven (PW) glass fiber mats with comparable volume fractions of traditional lay-up techniques, but significant higher delamination resistance. Fiber plies were manually introduced into the printing system and printed as typical print layers to form a composite part. The DLP method offers several advantages over traditional hand lay-up techniques, such as selective spatial reinforcement of a part, control over interlayer spacing, and the use of multiple resins in a single part [21].

In this work, we utilize the abovementioned AM technique to determine the effect of RRL thickness and resin properties on composites made with chopped strand glass fiber (CSGF) mats. The method is used to control the interleaf spacing, i.e., RRL thickness, and the resin material in flat composite structures that could not be achieved with traditional manufacturing methods. This offers a unique opportunity to study design parameters of interleaved composites. Unique composite test specimens were prepared and tested in short beam shear (SBS), flexure, tensile, and mode II delamination. Interestingly, we show that there are significant differences in the trends for CSGF mats and PW fabrics for varying RRL thickness. Furthermore, this work examines the important printer design criteria for achieving high fiber volume fractions of CSGF. Although CSGF is utilized in many applications for its low cost and isotropic properties, the random orientation of the fiber mats lead to significant processing issues that must be overcome to achieve successful consolidation. This work quantitatively demonstrates the issues with wet fiber mat consolidation using AM, and discusses engineering solutions.

## 2. Materials

This work primarily utilized a benchmark additive resin called DA-2, previously discussed in [24], which is composed of: bisphenol A glycidyl methacrylate ‘Bis-GMA’ (37.5 wt%), ethoxylated bisphenol A dimethacrylate ‘Bis-EMA’ (37.5 wt%), 1,6-hexanediol dimethacrylate ‘HDDMA’ (25 wt%), and a photoinitiator, Phenylbis (2,4,6-trimethylbenzoyl) phosphine oxide ‘PPO’ (0.7 wt%). Tenacious resin was purchased from Siraya Tech and used as received. The reinforcing material was randomly oriented glass fiber, purchased from Orca Composites. Properties of the resins and fiber are provided in Table 1 and Table 2, respectively.

## 3. Experimental Methods

**Figure 1:**
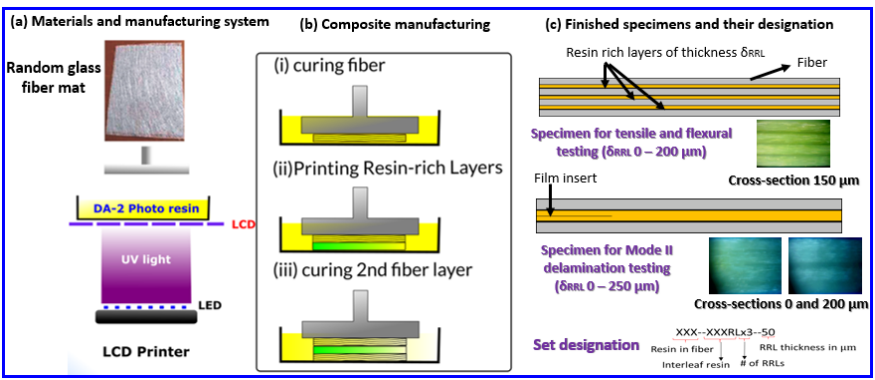
Demonstration of RRL incorporation in 3D-printed CSGF composite bars.

Figure 1 below demonstrates the printing process and shows images of RRLs in the final printed parts.

### 3.1. Composite Bars Printing

An ELEGOO MARS LCD printer (405 nm light) was used to produce all composite specimens. In all cases, a neat layer of resin was printed initially to ensure good quality surface finish, good adhesion to the build platform, and ease of sample removal. Subsequently, the platform was sent to a raised position such that the first fiber ply was added by hand to the resin vat. Note that the fiber ply is pre-wetted with resin to minimize void fraction in the final 3D printed composite. The building platform was then sent to full down position to consolidate the fiber mat. Once consolidated, the LCD screen shined blue light through the bottom of the vat for a determined exposure time to selectively cure the resin in the fiber mat. Once the resin is cured, the building platform was raised and the printing paused to ensure that the cured fiber ply adhered well to the building platform, and any excess fiber along the print edges were removed. The latter step is important to ensure that excess fiber does not prevent the build platform from pressing down on subsequent mats with a uniform pressure. All fiber layers are composed of four mats. Thus, the above process is repeated four times until a fiber-reinforced section was completed. Once completed, either another fiber layer was added or an RRL was printed. An RRL with a desired thickness was added by zeroing the printer height with the printed specimen and then printing resin-only layers until the desired RRL thickness was achieved. This process was utilized to print tensile and flexural specimens/bars with three RRLs and four fiber-reinforced sections. Selected $\delta_{RRL}$ values are 50, 100, 150, and 200 µm. Two more sets for tensile and flexural testing were manufactured via vacuum-assisted resin transfer molding (VARTM) to compare their properties with the 3D-printed specimens. Mode II delamination test specimens were printed with only one central RRL ranging in thickness from 0 to 250 µm (50 µm increments), and a film insert was introduced in the mid-plane of the RRL for the Mode II delamination studies. Additionally, 0-µm RRL specimens were printed for tensile, flexural, and Mode II delamination testing to determine the effect of $\delta_{RRL}$ on mechanical properties, as well as a short beam shear (SBS) testing set. All sets tested with their respective dimensional measurements are provided in Table 3. Microscopy imaging of the individual interleaved specimens show that resin layers were printed with good dimensional accuracy; their details are provided in Table 4.

### 3.2. Composite Post-Processing

All printed parts underwent a post-curing procedure inside a Formlabs 405 nm UV light oven (Somerville, MA) at a temperature of 75 °C for 2 h. Afterwards, the bars were polished to remove excess fiber on the edges and also polish the faces of the specimens to minimize defects that could impact the mechanical testing results.

### 3.3. Testing Conditions

#### 3.3.1. Short Beam Shear (SBS) Testing

SBS testing was conducted on rectangular specimens following standard ASTM D2344/D2344M-16 [26], where the cross-head speed was set to 1 mm/min, the span length-to-thickness ratio was kept constant at 2:1, and the SBS stress was determined from:

(1) $$\sigma_{\mathrm{SBS}}=\frac{3P}{4bh}$$

where *P* is the load measured and *b* and *h* are the specimen’s width and thickness, respectively.

#### 3.3.2. Flexure Testing

Flexural testing was conducted on rectangular bars according to standard ASTM D790-17 [27], where the cross-head speed was set to 1 mm/min, and the span length-to-thickness ratio was kept constant at 16:1. Note that all test specimens were composed of two fiber ply regions (top and bottom) separated by one RRL. Flexural strength and modulus were calculated using the equations below:

(2) $$\begin{array}{c} \sigma_{f}=\frac{3PL}{2bd^{2}} \end{array}$$

(3) $$\begin{array}{c} E_{B}=\frac{mL^{3}}{4bd^{3}} \end{array}$$

where *P* is the measured load, *L* is span length, *m* is the slope of the linear portion of the load-displacement curve, and *b* and *d* are the specimen’s width and thickness, respectively.

#### 3.3.3. Tensile Testing

Tensile testing was conducted on rectangular bars following the structure ASTM D3039/3039M-17 [28]. Cross-head speed was set to 0.5 mm/min, gauge length was 50 mm, and the applied gripping pressure was 350 psi. Mechanical testing was performed using an MTS 370.10 servo hydraulic frame equipped with a 100 kN load cell. Specimen displacement and strain were measured by using a 3D Digital Image Correlation (DIC) system comprising of two 5 megapixel Baumer TXG50 monochrome cameras with 2/3″ Charged Coupled Device (CCD) sensors (manufactured by GOM-3D Metrology). A field of view of 55 × 44 mm^2^ was chosen, while image capturing was performed at a rate of 0.2 Hz. The deformation measurements were obtained using the subset method with 40 × 40 pixels facet size and 20 pixels step size. Tensile strength, modulus, and strain were determined using:

(4) $$\begin{array}{c} \sigma_{T}=\frac{P_{max}}{A} \end{array}$$

(5) $$\begin{array}{c} \epsilon =\frac{\delta }{L_{g}} \end{array}$$

(6) $$\begin{array}{c} E_{T}=\frac{\Delta \sigma }{\Delta \epsilon } \end{array}$$

where $P_{max}$ is the measured load before failure, *A* is the specimen’s cross-sectional area, δ is the measured displacement via an extensometer, $L_{g}$ is the gauge length, and Δσ/Δϵ is the initial slope of the stress strain curve.

#### 3.3.4. Mode II Delamination

Delamination testing in mode II was performed using a three point bending set up. The samples were printed with only one resin layer in the mid-plane with a given thickness in the range described in the previous section. A polyimide film of 40 mm in length was printed in the center of the RRL layer to initiate the crack. Specimen geometry and testing conditions were selected following ASTM D7905/D7905M standard [29]. The mode II interlaminar fracture toughness ($G_{IIc}$) was calculated using:

(7) $$G_{IIc}=\frac{3mP_{max}^{2}a_{0}^{2}}{2B}$$

where *m* is a parameter obtained from compliance testing, $P_{max}$ is the maximum load reached during delamination testing, $a_{0}$ is the position of the delamination test marking, which was chosen to be 30 mm from the insert’s end, and *B* is the specimen’s width.

### 3.4. Fiber Volume Fraction (FVF) and Void Fraction Measurement

The true volume fraction of the printed specimens was experimentally determined via ignition loss experiments following ASTM standard D2548-18 [30]. The specimens were weighed, $W_{b}$, and then heated to 600 °C and cooked for one hour. Specimens were weighed post heating, $W_{a}$, and the actual FVF, $\phi_{f,a}$, was calculated via:

(8) $$\phi_{f,a}=\frac{\frac{W_{a}}{\rho_{g}}}{\frac{W_{a}}{\rho_{g}}+\frac{W_{b}-W_{a}}{\rho_{r}}}$$

The void fraction of the specimens, $\phi_{v}$, was calculated using equation:

(9) $$\phi_{v}=1-\frac{\frac{W_{a}}{\rho_{g}}}{\frac{W_{a}}{\rho_{g}}+\frac{W_{b}-W_{a}}{\rho_{r}}}-\frac{\frac{W_{b}-W_{a}}{\rho_{r}}}{V_{c}}$$

where $V_{c}$ is the sample’s total volume.

## 4. Results and Discussion

### 4.1. FVF Results

**Figure 2:**
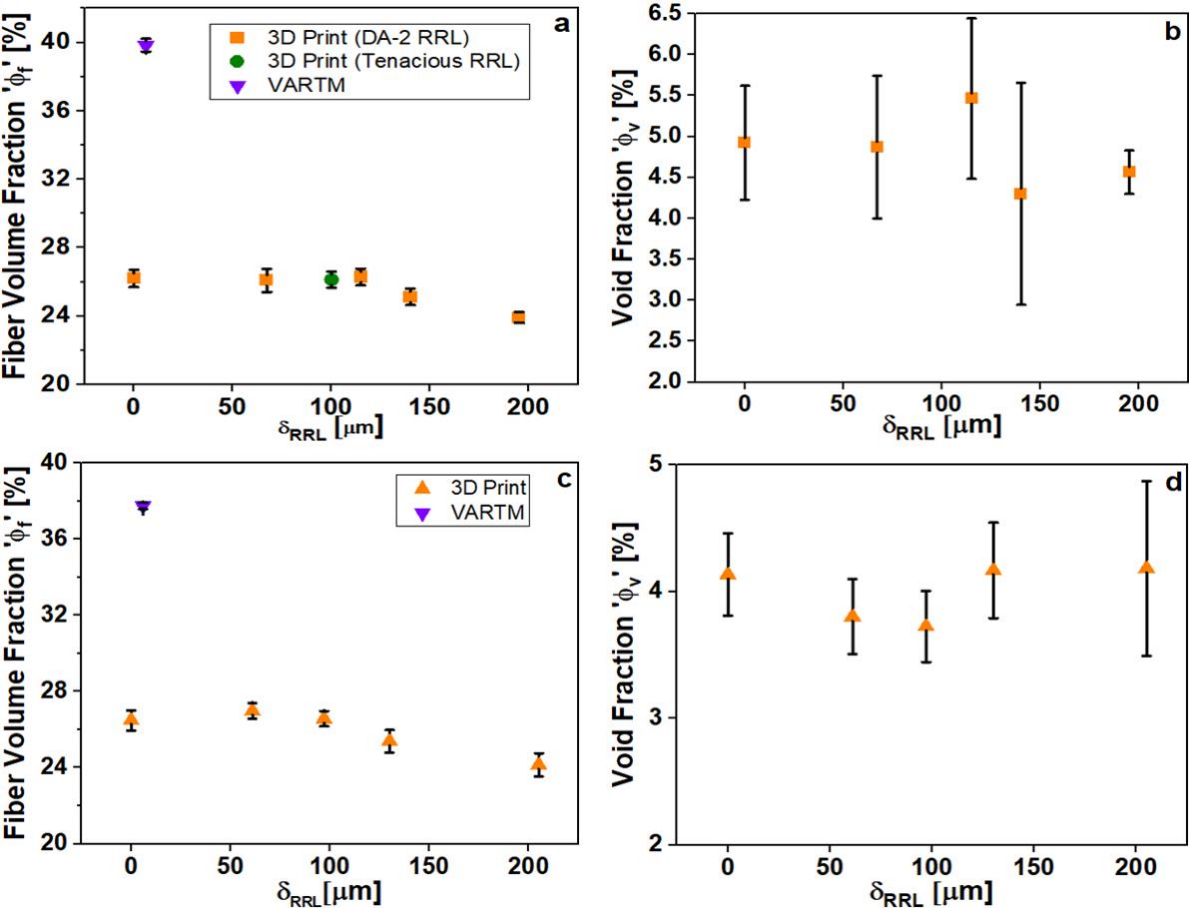
FVF measurements (**a**) and void fraction (**b**) for flexure specimens; FVF measurements (**c**) and void fraction (**d**) for tensile specimens.

The volume fraction of reinforcement material is directly related to the mechanical properties of the composite. In traditional manufacturing methods, there is a fiber consolidation step to increase the fiber volume fraction before curing. In the AM method here, the consolidation step is facilitated by the downward pressure of the build platform on the fiber mat prior to photo-cure. Figure 2 shows fiber volume and void fraction measured for flexural and tensile specimens as a function of RRL thickness. In Figure 2a,c, there are three important observations: (i) the AM specimens have much lower volume fractions than VARTM samples, (ii) the flexural and tensile specimens have FVF that does not decrease with RRL thickness for $\delta_{RRL}<125$ µm, and (iii) that the volume fractions for flexural and tensile specimens are very similar. Point (iii) is a testament to the reproducibility of this manufacturing method, while points (i) and (ii) were somewhat surprising and will be discussed in detail below.

Another important aspect of composite manufacturing is the introduction of voids during manufacturing [31,32]. The voids act as defects that can cause premature failure of the specimen. Vacuum-assisted methods are advantageous, as they minimize the amount of trapped air. The AM method is done in ambient conditions and thus void fraction is a reasonable concern. Figure 2b,d show the void fraction measured in the different test specimens printed using AM. While all void fractions are non-zero, the void fractions are relatively constant with increasing RRL thickness for tensile and flexural specimens. This suggests that all voids are coming from the process of introducing the fiber mat. The large error bars indicate large variations in void distribution for different printed specimens. Note that the introduction of a dry fiber mat into the resin vat introduced significantly higher void fractions (data not shown). Thus, pains were taken to pre-wet the fiber mat before introduction into the resin vat to avoid the trapping of air bubbles during mat placement. Regardless of these efforts, the void fraction for the specimens could not be reduced below 4–5%. To minimize the void fraction further, one could imagine introducing a fiber pre-wetting step that is carried out under vacuum conditions, which is currently in the works.

#### 4.1.1. Physics of Mat Consolidation

As fiber volume fraction is arguably the most important parameter in determining mechanical properties, we investigated the reasons behind the lower volume fraction between AM and VARTM, and the lack of decrease in FVF below $\delta_{RRL}<125$ µm. There are two reasons for the lower FVF in AM specimens compared to VARTM: (1) lower applied pressure during consolidation and (2) the larger pressure required to consolidate a wet mat versus a dry mat. In the case of (1), the z-motor in a DLP printer has a finite amount of torque, which limits the applied pressure. The maximum downward force measured on the printer was 150 N, which gives a maximum consolidation pressure of 0.03 MPa considering a 110 mm by 45 mm fiber mat. In VARTM, the pressure is uniform and equal to 0.1 MPa everywhere.

**Figure 3:**
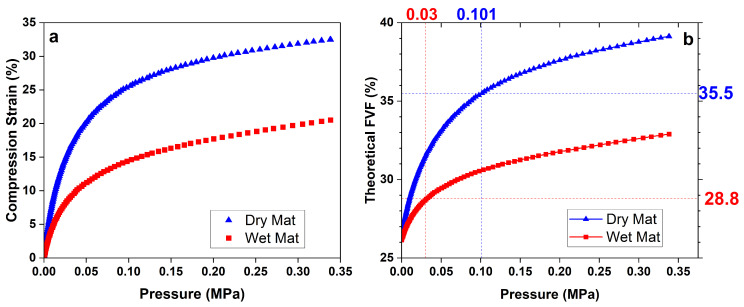
Fiber compression results and fiber volume fraction prediction.

A simple experiment was conducted to demonstrate this point more clearly. Figure 3a shows a compression strain as a function of pressure for a single dry and wet fiber mat measured using a parallel plate geometry on an Instron machine via a moving top-plate and stationary bottom plate. The compression strain for a given downward force was measured via a change in height of the top plate accounting for compliance of the machine. The theoretical fiber volume fraction can be calculated via a modified Composites Research Advisory Group (CRAG) Equation [33] given by:

(10) $$\phi_{f,theoretical}=\frac{\rho_{areal}}{\rho_{g}}\frac{n_{plies}}{h_{dry}\left(1-\epsilon_{compression}\right)}$$

where $\rho_{areal}$ is the areal weight of the fiber mat and $\epsilon_{compression}$ is the measured compression strain of the fiber mat. Figure 3b shows the calculated FVF using Equation (10) as a function of consolidation pressures. This graph clearly shows that a dry fiber mat is capable of achieving a little less than twice the FVF of a consolidated wet mat at the same pressure. This important result shows the difficulty in achieving high FVF of random chopped fiber mats with AM methods.

Taking into account the different pressures applied by the VARTM and AM, the consolidation experiment predicts a FVF in AM of 28–29% and 36% in VARTM; see the dotted lines on Figure 3b. Recall from Figure 2a that VARTM and AM achieved a FVF of 40% and 26%, respectively. Overall, the theoretical predictions via compliance are very close to the experimentally determined values in both cases, which supports the argument that the consolidation of wet random chopped glass fiber mats in AM requires significantly higher pressures/forces than VARTM. Figure 3 clearly shows that FVF of AM CSGF composites can be increased by using higher torque motors in the z-axis to increase pressure. However, one must be cognisant that additional pressure could lead to fiber breakage, limiting the amount of pressure allowed. This work is currently under way.

The fact that VARTM resulted in higher mat consolidation than the theoretical prediction in Figure 3 can be explained by the fact that VARTM performs its consolidation with uniform pressure compared to the Instron and AM methods, which apply pressure via a solid platform and are, thus, subject to stress variations. Unfortunately, this issue cannot be overcome in AM methods, and thus the theoretical wet mat consolidation curve is expected to hold. A more accurate dry mat consolidation theoretical curve could be achieved using VARTM at various vacuum pressures. However, the dry mat results in Figure 3 offer a lower limit and suffice to point out the differences between the VARTM method and AM method. Interestingly, there was very little discrepancy in VARTM and AM FVF with PW mats due to the fact that they require much less consolidation [21].

The last result that needs explaining is the unchanged FVF with increasing RRL thickness for $\delta_{RRL}<125$ µm. Ideally, one would have expected to see that the inclusion of resin-rich domains would decrease the FVF of the specimen proportionately. However, the fact that the FVF stays constant for relatively small $\delta_{RRL}$ means that the consolidation of the fiber mat is dependent on RRL. In other words, the only explanation for a constant FVF with larger resin-rich domains is that the fiber mat layers have increasing FVF with increasing RRL thickness. This can only mean that the presence of an RRL increases the consolidation of the subsequently printed fiber mat. One reason for the better mat consolidation is that the RRL provides a more compliant surface by which to apply pressure to the subsequent mat layer. In other words, the mechanical properties of the layer just before mat consolidation is important in ensuring a uniform distribution of stress when the build platform compresses the mat. This dependence should be taken into account when designing an automated AM method of mat placement and consolidation. We now look at how the mechanical properties of AM composite specimens depend on RRL thickness.

### 4.2. Short Beam Shear/Interlaminar Shear Strength

**Figure 4:**
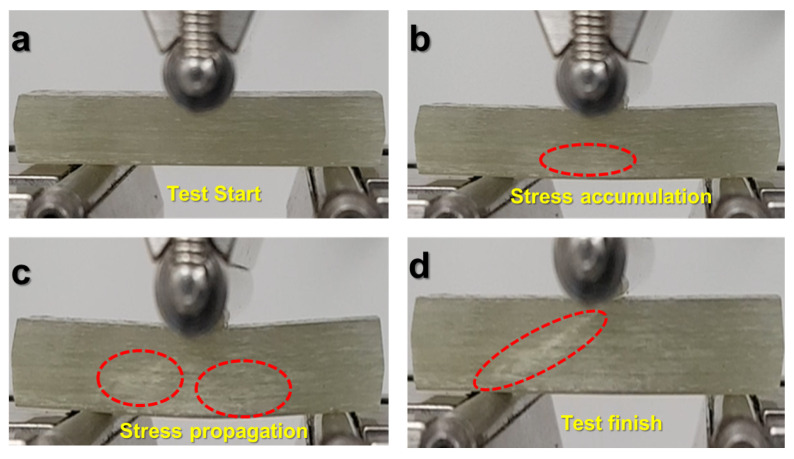
(**a**) SBS test start. (**b**) Cracking signs appearing due to accumulated stress. (**c**) Crack and stress propagation. (**d**) SBS test finish.

**Figure 5:**
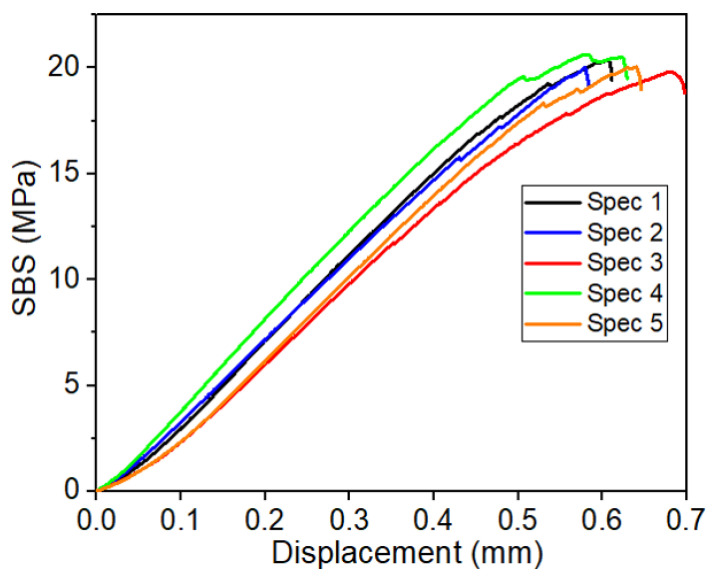
SBS-displacement curves for the tested specimens.

SBS test was conducted to evaluate the contact strength between CSGF and DA-2 resin (i.e., interlaminar shear strength). During this test, the loading roller applied a compressive force on the beam, leading to formation and propagation of cracks at the center of the specimen from the bottom to the top, illustrated by Figure 4a–c. Note that the test was stopped upon the recording of first load drop (Figure 5), corresponding to diagonal crack formation as in Figure 4d, as per ASTM standard D2344/D2344M-16, and for facilitation of understanding the composite’s deformation behavior. Figure 5 shows SBS versus displacement curves for five specimens.

The tested specimens did not show any delamination failure. This was discussed in the work by [34,35], where they attributed the observed specimen damages to mixed shearing and compressive buckling caused by the loading roller. These results indicate that stress distribution through the thickness of the SBS specimen deviates from classical beam theory, where the stress is expected to be highest at beam’s mid-plane. The reason for this discrepancy is unknown and is still being investigated. From the curves in Figure 5, the measured SBS for DA-2/CSGF composites was 20 MPa, which is remarkably higher than the literature values (i.e., 10–15 MPa) of VARTM epoxy composites using the same fiber mat [36]. This finding confirms the good adhesion and contact between DA-2 and CSGF.

### 4.3. Static Mechanical Properties Testing

#### Flexural and Tensile Testing

**Figure 6:**
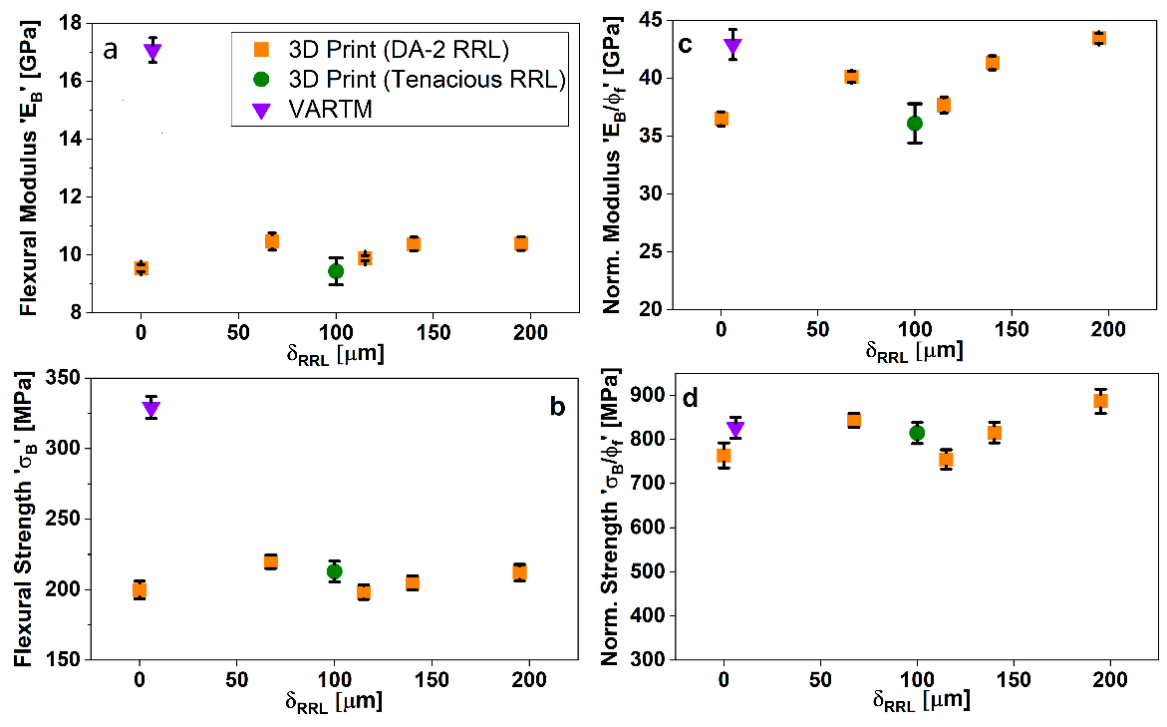
Flexural properties comparison with respect to RRL thickness.

Figure 6 shows the flexural properties measured for different $\delta_{RRL}$ and two different resins, DA-2 and Tenacious (see Table 1 for material properties), compared to a set made using a traditional composite manufacturing technique, VARTM. Unlike the case of PW fiber fabric reported in Idrees et al., the VARTM modulus and strength results are considerably higher than the 0-µm RRL samples printed with CSGF mats [21]. This difference can be explained by the very different FVF that is achieved using VARTM versus AM, see Figure 2. This is discussed in depth in Section 4.1.1.

Figure 6a,b show a relatively constant modulus and strength with increasing $\delta_{RRL}$, despite the drop in FVF at > 100-µm $\delta_{RRL}$. As explained above, a thicker RRL is more efficient in applying uniform pressure on the imperfect mat landscape than a hard reinforcing fiber layer. Thus, the laminate layers have an increased modulus in the fiber-reinforced zones, which contributes to the overall increase in composite modulus due to the presence of RRLs. This is further validated by specimens printed with a Tenacious RRL. The Tenacious RRL specimens have almost identical modulus and strength to the pure DA2 specimens. This should not be the case given the lower modulus and strength of Tenacious; see Table 1. Again, this can only be explained by an increased consolidation of the mat layer by the inclusion of a softer more compliant RRL. The data strongly suggest that the additive manufacturing of GFRC using DLP should consider RRL for improved laminate consolidation Note that this is not the case for oriented fiber mats, as described in Idrees et al. [21], where consolidation is not so important. Figure 6c,d show the modulus and strength normalized by the FVF. We can see from the normalized properties that the non-monotonic behavior is exaggerated and is different to the constant trend observed for oriented fiber mats [21].

**Figure 7:**
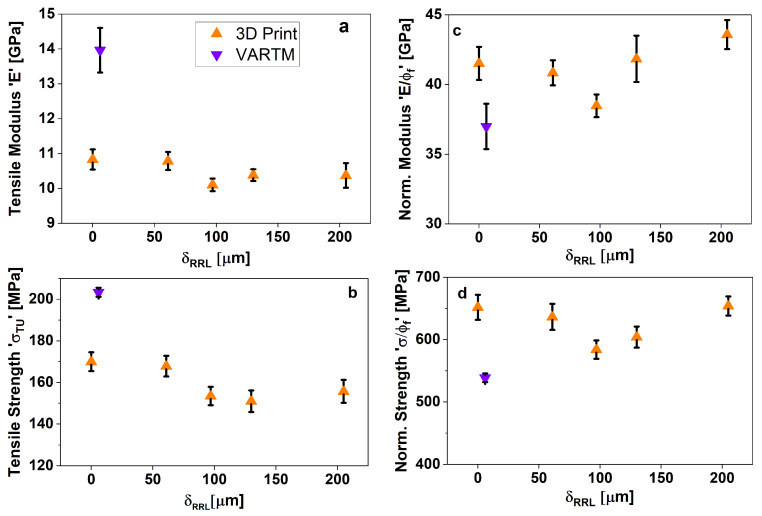
Tensile properties comparison with respect to RRL thickness.

As in flexural testing, two batches were made for testing tensile properties of CSGF DA-2 composites, one 3D printed and one VARTM. Figure 7a,b show that the tensile modulus remained almost unchanged for all $\delta_{RRL}$, whereas the strength slightly declined for $\delta_{RRL}=100$ µm, but remained constant for larger thicknesses. Overall, the trends between flexural and tensile specimens are very similar. The normalized properties shown in Figure 7c,d clearly highlight the non-monotonic behavior of the random fiber mat composites with increase in $\delta_{RRL}$, reflecting the effect of laminate consolidation on the observed trends. Interestingly, Figure 7c,d show that the normalized properties of all AM specimens are higher than the VARTM processed samples.

### 4.4. Mode II Delamination

**Figure 8:**
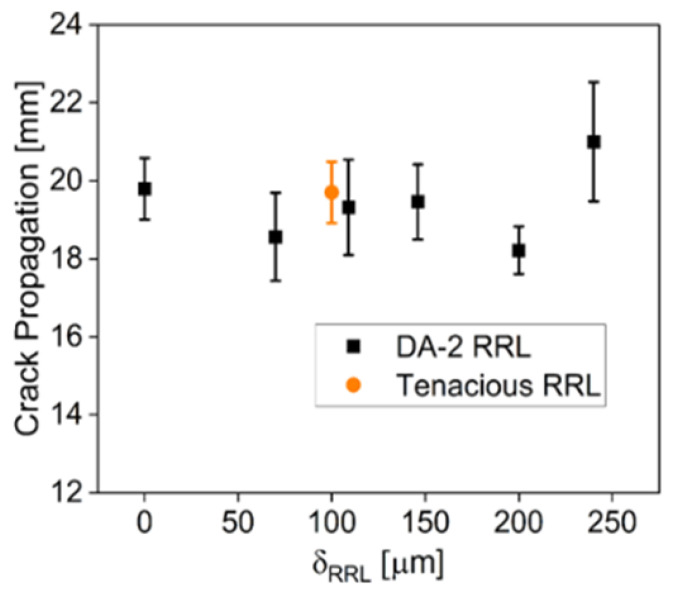
Crack propagation versus $\delta_{RRL}$.

Mode II delamination was used to quantify the effect of RRL thickness on the delamination resistance, i.e., fracture toughness. Note that mode II measures delamination resistance under predominately shear loading. For mode II delamination testing, six sets were made with $\delta_{RRL}$ ranging from 0 to 250 µm using DA-2 as both fiber matrix and RRL. An additional 100 µm RRL thickness set was printed using DA-2 as the fiber matrix and the tough resin, i.e., Tenacious, for the RRL. The propagation of the crack was monitored during the test and the crack for all samples tested was observed to initiate at a displacement between 2.5 and 3 mm. Furthermore, Figure 8 shows that the final crack propagation length was relatively independent of the RRL thickness and resin chemistry. The only major difference between samples was the load required to initiate and propagate the crack.

**Figure 9:**
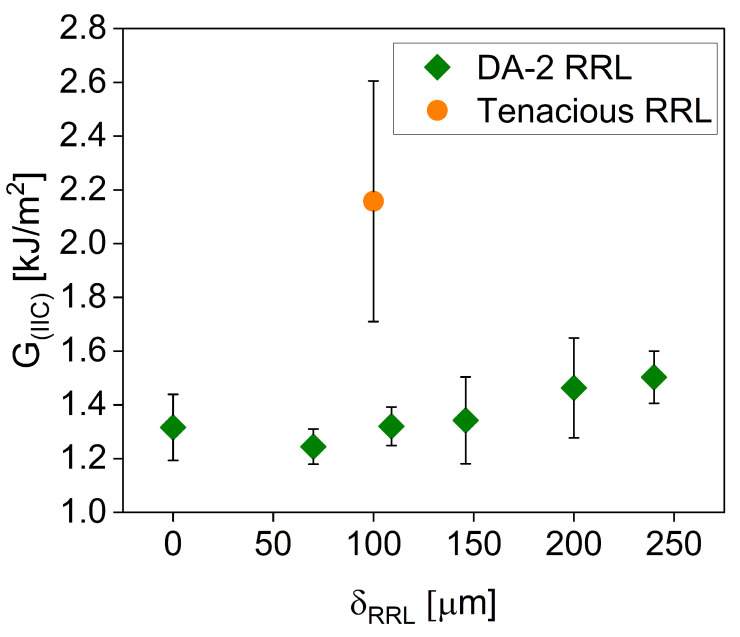
$G_{IIc}$ versus $\delta_{RRL}$.

Figure 9 shows that the $G_{IIc}$ is a weak function of RRL thickness using DA-2. The magnitude of $G_{IIc}$ is independent of RRL thickness up to $\delta_{RRL}=150$ µm, and only slightly increases at 200 and 250 µm. This very small increase in delamination resistance with $\delta_{RRL}$ is below that of other published works, where $\delta_{RRL}$ showed $G_{IIc}$ improvement upwards of 60% [11,13]. Thus, these results underline the importance of the RRL chemistry. This is further exemplified in the use of Tenacious resin in the RRL, which has a profound impact on the $G_{IIc}$, i.e., almost a two-fold increase in $G_{IIc}$. Furthermore, these results are for the most part consistent with a previous study using DA2/PW GF woven fiber [21], except for some key differences. For example, unlike DA2/PW GF composites, CSGF mats show no clear correlation between the peak load and overall crack propagation. Another important difference was the higher $G_{IIc}$ values for CSGF composites compared to PW composites. The average $G_{IIc}$ value was 0.94 kJ/m^2^ for 0 µm RRL using PW fabric, whereas the average $G_{IIc}$ value was 1.317 kJ/m^2^ using CSGF mats. Although in both fiber materials, the measured $G_{IIc}$ decreases for $\delta_{RRL}$ = 50 µm, the decrease using CSGF mats was less sharp, i.e., 6% from 1.317 to 1.245 kJ/m^2^ compared to 46% from 0.94 to 0.5 kJ/m^2^. Moreover, CSGF mats showed an increasing $G_{IIc}$ with $\delta_{RRL}$ above the $G_{IIc}$ of $\delta_{RRL}$ = 0 µm, while PW fiber composites showed the highest $G_{IIc}$ measured at $\delta_{RRL}$ = 0 µm.

One explanation for the differences between woven fabric and random mats is the different FVF. More specifically, the PW composites were printed with FVF around 40%, compared to the approximately 27% presented here. Davies et al. showed that the delamination resistance decreases with increasing FVF [37]. However, more work is needed to better understand the importance of mat architecture and FVF on the overall delamination resistance of interleaved composites.

## 5. Conclusions

This paper demonstrated successful fabrication of interleaved random glass fiber-reinforced thermosetting polymer composites via DLP, a highly promising vat photo-polymerization AM technique. The key advantage of this technique is efficient and accurate control over interleaf location, chemistry, and dimension. Note that the specimens presented here could not be easily achieved with traditional manufacturing methods. While there are clearly many advantages to the presented AM composite manufacturing process, there exist several challenges that must be considered. Namely, random chopped glass fiber is not easily consolidated using the AM build platform due to the higher pressures required compared to VARTM. This work clearly demonstrated the quantitative differences in the consolidation of wet and dry fiber mats, which is an important consideration when determining FVF of printed parts. Furthermore, it was not possible to eliminate void defects in this process. New strategies would need to be developed to avoid the inclusion of air pockets during the AM composite manufacturing process, such as a vacuum chamber.

The AM composite manufacturing process was used to to study the effect of RRL thickness and chemistry on the tensile, flexure, and interlaminar toughness of printed composite specimens. From the obtained results, we concluded the following:

1. DA-2/CSGF composites exhibit remarkably higher intelaminar shear strength than other same fiber composites reported in the literature.
2. Additively manufactured random glass fiber composites are about 50% lower in FVF than VARTM composites due to printer motor limitations and the much higher pressures needed to consolidate pre-wetted fiber mats.
3. The presence of RRLs increases fiber mat layer consolidation by distributing the applied consolidation stress more evenly across the mat.
4. Interleaving using brittle resins does not significantly increase mode II delamination resistance. However, significant increases are observed when a ductile resin was used for the RRL. Thus, the resin used for interleaving strongly determines the overall interlaminar fracture toughness of the part.
5. DA-2/CSGF composites have higher Mode II delamination resistance than woven glass fiber composite parts. However, this difference could be due to the very different FVF.

Overall, additive manufacturing is a reliable method for incorporating RRLs in composite parts using a layup technique. The layup process could be automated and incorporated into stereolithographic methods for the facile production of stiff, tough parts using multiple resins and selective incorporation of interleaved domains. More work is required to understand the optimum resin properties for the interleaf and the limitations on toughness and failure mechanisms. These questions are the subject of ongoing investigations.

## Figures and Tables

**Table 1:** Properties of DA-2 and Tenacious.

Property	DA2	Tenacious
Density (25 °C)	1.20	-
Glass Transition Temperature [$T_{g}$] (°C)	99	41.97
Tensile Modulus (GPa)	2.80 ± 0.10	1.9 ± 0.09
Tensile Strength (MPa)	61.9 ± 6.30	37.3 ± 2.80
Tensile Strain at Failure (%)	2.5 ± 0.60	44.06 ± 1.90
Flexural Modulus (GPa)	3 ± 0.10	1.6 ± 0.20
Flexural Strength (MPa)	110 ± 10	51 ± 5.20
Fracture Toughness [$G_{Ic}$] (J/m^2^)	58.80 ± 0.30	1580

**Table 2:** Properties of glass fiber.

CSGF Properties	
Areal Weight (g/m^2^ or gsm)	900
Density (g/cm^3^ or g/cc)	2.68 [25]
Tensile Strength (MPa)	3100–3800 [25]
Tensile Modulus (GPa)	80–81 [25]
Elongation at Break (%)	4.5–4.9 [25]

**Table 3:** Specimen dimensions for mechanical testing, see Section 3.3 for respective ASTM standards.

Manufacturing Method	Test	Length (mm)	Width (mm)	Height (mm)
3D Printing	Short beam shear (SBS)	40	11.50	6.40–6.70
	Tensile	100	12	3.90–6.20
	Flexure	110	12.35	4.00–6.60
	Mode II delamination	110	19	4.30–6.00
VARTM	Tensile	100	12	2.80–2.90
	Flexure	110	14	2.85–3.10

**Table 4:** RRL measurements for interleaved sets.

Test	Set	Average Measured $\delta_{\mathrm{RRL}}$ (µm)
Tensile	DA2-DA2RLx3-50	67.28 ± 2.09
	DA2-DA2RLx3-100	114.67 ± 4.22
	DA2-DA2RLx3-150	140.27 ± 4.99
	DA2-DA2RLx3-200	195.08 ± 8.41
Flexure	DA2-DA2RLx3-50	61.33 ± 1.28
	DA2-DA2RLx3-100	97.29 ± 2.34
	DA2-DA2RLx3-150	128.43 ± 1.57
	DA2-DA2RLx3-200	205.43 ± 5.46
	DA2-TENRLx3-100	109 ± 2.5
Mode II Delamination	DA2-DA2RLx1-50	70 ± 2.89
	DA2-DA2RLx1-100	108 ± 6.90
	DA2-DA2RLx1-150	146 ± 8.72
	DA2-DA2RLx1-200	200.4 ± 6.45
	DA2-DA2RLx1-250	240 ± 9.76
	DA2-TENRLx1-100	111 ± 5.00

## Data Availability

Data are available upon request.
